# Construction of a high-density interspecific (*Lens culinaris* x *L*. *odemensis*) genetic map based on functional markers for mapping morphological and agronomical traits, and QTLs affecting resistance to *Ascochyta* in lentil

**DOI:** 10.1371/journal.pone.0214409

**Published:** 2019-03-27

**Authors:** Carlos Polanco, Luis Enrique Sáenz de Miera, Ana Isabel González, Pedro García, Richard Fratini, Francisca Vaquero, Francisco Javier Vences, Marcelino Pérez de la Vega

**Affiliations:** Área de Genética, Departamento de Biología Molecular, Universidad de León, León, Spain; Università Politecnica delle Marche, ITALY

## Abstract

Usage of high-throughput sequencing approaches allow for the generation and characterization of reference transcriptome datasets that support gene-based marker discovery, which in turn can be used to build genetic maps among other purposes. We have obtained a transcriptome assembly including 49,453 genes for the lentil (*Lens culinaris* Medik.) cultivar Alpo using RNAseq methodology. This transcriptome was used as reference to obtain 6,306 quality polymorphic markers (SNPs and short indels) analyzing genotype data from a RIL population at F_7_ generation derived from the interspecific cross between *L*. *culinaris* cv. Alpo and *L*. *odemensis* accession ILWL235. *L*. *odemensis* is a wild species included in the secondary gene pool and can be used as a source for gene introgression in lentil breeding programs. Marker data were used to construct the first genetic interspecific map between these two species. This linkage map has been used to precisely identify regions of the CDC-Redberry lentil draft genome in which the candidate genes for some qualitative traits (seed coat spotting pattern, flower color, and stem pigmentation) could be located. The genome regions corresponding to a significant single quantitative trait locus (QTL) controlling “time to flowering” located in chromosome 6 and three QTLs regulating seed size and positioned in chromosomes 1 and 5 (two QTLs) were also identified. Significant QTLs for *Ascochyta* blight resistance in lentil were mapped to chromosome 6 in the genome region or close to it where QTLs for *Ascochyta* blight resistance have previously been reported.

## Introduction

In agriculture, the main goal of hybridization between crops and their wild relatives (wide-crosses) is the introgression of adaptive traits from the wild relatives into the cultivated forms as part of breeding programs [1]. Wide-crosses have extensively been used in plant breeding to broaden the gene pool of many crop species and to transfer desirable particular genes, mainly resistance genes to biotic or abiotic stresses. Response to biotic stresses is the main reason alleged to use wild crop relatives in breeding programs among the 10 main crops [2]. Wild germplasm provides the raw material used for resistance breeding in what is designated as the “smart breeding” strategy which facilitates exploration and utilization of the available natural genetic variation, thus expanding the genetic base of crop plants while providing more adaptive flexibility with respect to the future [3,4]. Wide-crosses implicate not only the primary gene pool, that is the wild form of the cultigen together with other species producing fertile hybrids, but also the secondary and tertiary gene pools. Wide-crosses have largely been used in cereal breeding [5–9], primarily in wheat (resistance has been introgressed from at least 52 species from 13 genera [10]), barley and rice, and in the dicot species tomato [11]. Although less extensively used than in cereal species, wide-crosses have also been used in breeding legume species [12–15]. Usually, wide-hybridization depends upon embryo rescue techniques to obtain interspecific hybrids since specific isolation often results in weak or completely unviable embryos. Thus, availability of efficient embryo rescue protocols based on *in vitro* culture techniques remains a bottleneck to systematic use of interspecific hybridization applied to breeding purposes [16].

Lentil (*Lens culinaris* Medik. subsp. *culinaris*) is a diploid (2n = 14), self-pollinating annual cool season grain legume normally grown in temperate semi-arid regions, usually in rotation with cereals. Lentils are traditionally valued as a source of energy, proteins and iron in human nutrition. In addition, they are an important dietary source of fiber, minerals, vitamins and antioxidants. Likewise, lentils contribute to improve soil quality by replenishing soil nitrogen levels and can play a significant role in environmentally sustainable agricultural systems. The crop is now extensively cultivated throughout Western Asia, Northern Africa, the Indian subcontinent, Australia and North America, in particular in Canada as the current first world producer and exporter [17,18]. Wild lentil relatives are being broadly used to develop genetic maps, to analyze quantitative trait loci (QTLs) related to yield and other plant characters, as a source of resistance genes to both biotic and abiotic stresses, and in general to broadening the genetic base of lentil cultivars [19–29]. Singh et al. [30] have recently reviewed and summarized the available wild *Lens* germplasm, the characterization and evaluation of wild species for useful traits of interest to solve production-related problems, as well as the progress achieved to widen the genetic base of the cultivated lentil varieties through wide hybridization and explorative lentil genomics.

Interspecific *Lens* seeds most often fail to reach full development or else are unable to germinate, therefore *in vitro* embryo-rescue techniques are usually required to obtain hybrid plants between *Lens* species [31,32]. In a genotype-by-sequencing (GBS) characterization of *Lens* species, Wong et al. [33] included in the primary gene pool, in addition to the lentil cultigen, the wild ancestor *L*. *c*. subsp. *orientalis* (Boiss.) Ponert and the species *L*. *tomentosus* Ladiz., the secondary gene pool included *L*. *odemensis* Ladiz. and *L*. *lamottei* Czefr., in the tertiary *L*. *ervoides* Grande, and in the quaternary *L*. *nigricans* (M. Bieb.) Godr., while Ladizinsky and Abbo [34] included *L*. *odemensis*, *L*. *ervoides* and *L*. *tomentosus* in the secondary gene pool, and *L*. *nigricans* and *L*. *lamottei* in the tertiary.

Genetic maps based on recombination frequency distances between genes are classical elements of the formal genetic analysis, allowing researchers to locate genome regions of interest, hence remaining key breeding tools. The new high throughput technologies in genomics and transcriptomics provide an unprecedented level of insight into the structural diversity across crop genomes and afford thousands of genetic markers, therefore allowing to obtain highly saturated genetic maps providing a better location of QTLs and other genes of interest. Genetic maps based on markers derived from high throughput sequencing techniques have been developed in lentil [35,36,19,37,38,24]. Several reviews on lentil genetic maps obtained from morphological to molecular markers have been published [18,36]. Excluding intersubspecific maps between the cultivated lentil and its wild ancestor (*L*. *c*. subsp. *orientalis*), true interspecific genetic maps between the cultivated lentil and other *Lens* species have so far been limited to *L*. *ervoides* [24].

Introgressed chromosome segments from alien species often include favorable genes that result in selection, structural rearrangements that hinder or prevent recombination in the affected chromosomal segments, or both. As a consequence, sets of relatively close alleles from the same parent tend to remain together from generation to generation and frequently lead to segregation distortion; which in turn can decrease the capacity to determine correct marker orders in genetic linkage maps while impeding accurate QTL detection [39]. Different allele selective values generate linkage drag, the unintentional co-selection of undesirable gene variants that are closely linked to selected loci of interest [40]. High-resolution genome analysis technologies enable description of crop diversity at an unprecedented chromosome-scale resolution and they are highly suited for genomic selection strategies. Likewise, genome-wide diversity data sets enable the dissection of linkage disequilibrium to characterize loci underlying selective sweeps. On the other hand, high-density genome data may help to increase the use of distant gene pools by enabling more efficient genomic selection strategies that efficiently predict performance based on genome wide marker combinations [40].

Chromosomal rearrangements between *Lens* species have been described based on karyotype and genetic mapping analyses [41–43]. Differences between *Lens* species karyotypes using fluorescent in situ hybridization (FISH) techniques have been identified [44–46]. Furthermore, Galasso [46] using highly repeated DNA sequences established a typical FISH karyotype for each *Lens* species with very little variation within species, except in the case of *L*. *odemensis* in which a karyotype polymorphism was found.

We present here the first genetic interspecific map between *L*. *culinaris* and *L*. *odemensis*, from the primary and the secondary gene pools [33] respectively, based on 6,306 SNPs and short indels. Markers located in expressed genes that were genotyped in both parents and in an F_7_ RIL population which included a total of 78 lines. The genetic map was compared with the draft assembly of the *L*. *culinaris* (cv. CDC-Redberry) genome [47] and was used for mapping morphological and agronomical traits as well as QTLs related to resistance against the pathogen *Ascochyta lentis*.

## Materials and methods

### Plant and fungal materials

A bi-parental RIL population was developed from a single F_1_ hybrid plant derived from an interspecific cross between *Lens culinaris* cultivar Alpo and *L*. *odemensis* accession ILWL235, followed by single seed descent from the F_2_ population (120 F_2_ seeds were sown). A total number of 78 surviving RILs at the F_7_ generation were genotyped and phenotyped, as well as the two parental accessions. The *L*. *culinaris* cultivar Lupa was used as the susceptible control for inoculations with *Ascochyta lentis*.

A suspension of the *A*. *lentis* isolate AL-84 in a concentration of 1 x 10^6^ conidia ml^-1^ was used to spray-inoculate different replicates of three 15 day-old plantlets grown together under sterile conditions in capped glass tubes. Nine replicates of each of the two parental accessions and six replicates of each of the 78 RILs were inoculated. Another identical set of replicates per each line or parental sample was treated without the presence of the pathogen. Following 24 hours after the treatment, the aerial parts of the plantlets draw from six replicates of the pathogen-inoculated and the pathogen-free sets were collected independently for each parental accession and frozen for total RNA extraction. Three replicates of the pathogen-inoculated plants were collected and pooled for RNA extraction from each of the78 RIL lines. Total RNA was isolated using the method of Chang et al. [48]. The remaining replicates from the pathogen-inoculated and the pathogen free sets were used to score disease severity. Disease symptoms were checked 14, 21, and 28 days after infection [49]. There were no clear signs of infection after 14 days, either in the susceptible genotypes used to check the infection progression. The plants started to deteriorate after 28 days into capped glass-tubes, so the scores at 21 days were used in further analyses, using the 1–9 categorical scale described by Ford et al. [50].

The mapping population and the parental accessions were also reared under greenhouse conditions. Later they were visually phenotyped for morphological traits such as the presence of seed coat spotting (*Scp*), flower color (purple or white), and anthocyanin pigmentation on the plantlet stem (*Gs*), as well as the quantitative traits seed size in mm^2^ and time to flowering (scored as early, medium or late flowering). The average seed size was obtained from scanned images of 30 seeds using ImageJ software [51], and time to flowering was considered when 50% of the plants within a RIL had at least one flower [52].

### Libraries construction and Illumina sequencing

Total RNA quality and quantity of the samples to be sequenced was determined in the Bioanalyzer 2100 (Agilent Technologies, Santa Clara, CA, USA) and the Qubit 3.0 fluorometer (Thermo Fisher Scientific, Wilmington, DE, USA). The poly(A)+ mRNA fraction was isolated from the total RNA and strand-specific cDNA libraries were obtained following Illuminás recommendations. Briefly, poly(A)+ RNA was isolated on poly-T oligo-attached magnetic beads and chemically fragmented prior to reverse transcription and cDNA generation. The cDNA fragments then went through an end repair process, the addition of a single adenine base to the 3’ end followed by ligation of the adapters. Finally, the products were purified and enriched using PCR to create the indexed final double stranded and strand-specific cDNA libraries. The quality of the libraries was analyzed by the High Sensitivity RNA assay in a 4200 TapeStation (Agilent Technologies) and the quantity of the libraries was determined by real-time PCR using a LightCycler 480 (Roche Applied Science, Penzberg, Germany) according to the manufacturer’s protocols.

Twenty-four cDNA libraries were obtained using RNA from each of six replicates of both the inoculated and the non-inoculated plants from the two parental accessions. In addition, 78 cDNA libraries were obtained, one originating from each RIL line, using the pooled RNA from three inoculated replicates. Equimolar pools of cDNA libraries were sequenced by paired-end sequencing (100 x 2) in an Illumina Hiseq 2500 sequencer (Illumina Inc., San Diego, CA, USA).

### Transcriptome assembly, variant calling, and marker filtering

The quality of the raw sequence reads was assessed using the FASTQC tool [53]. The reads were filtered using Trimmomatic [54] with a sliding window size of 4 and leading and trailing parameters were set at a quality of 28. All the filtered reads obtained from the Alpo cultivar libraries were mapped after normalization onto the *L*. *culinaris* cv. CDC Redberry v1.2 genome assembly (http://knowpulse.usask.ca) using the STAR aligner [55]. A coordinate-sorted bam file was obtained using the SAMtools suite [56], which was used to generate a genome-guided *de novo* transcriptome assembly of the Alpo cultivar using the Trinity package [57] using the lentil v1.2 genome as reference. Duplicate and near identical contigs in the assembly were eliminated using the Dedupe BBTool from the Joint Genome Institute (University of California) with parameters e = 10 and minidentity = 95 in separate steps. The Magic-BLAST tool available online at the NCBI webpage was used to map each transcript onto the pseudomolecules (chromosomes) and contigs of the lentil v1.2 genome assembly.

Before SNPs and short indels calling, the abundance of the ALPO transcripts was quantified using the Kallisto program [58] in order to filter the transcripts to exclude isoforms that were lowly expressed. A Trinity script was used to retain the single most highly expressed isoform for each gene. Such transcripts were used as the reference for variant calling using SAMtools mpileup [56]. A single variant call file (VCF) file was generated from the 102 bam files that were previously obtained using the STAR aligner in order to map independently the filtered reads derived from each library onto the reference Alpo transcriptome.

The VCF variants were filtered for read depth and low quality using the mpileup software filter options. A custom Perl script was then used to retain high-quality variants analysing the genotype QUAL scores and to remove multiallelic variants, missing calls for 28 or more RILs, variants showing different genotypes in the parental replicates, or fixed variants in the RILs. Finally, a single VCF variant was selected for each transcript of the ALPO reference transcriptome.

### Genetic linkage mapping, comparative mapping, and QTL detection

The high-quality markers retained were used to construct a genetic linkage map using the MSTMap software tool [59], which determines the correct marker order by computing the minimum spanning tree of an associated graph, making use of the following settings: Kosambi’s distance function, cut-off p value = 1 x 10^−12^ for clustering the markers into linkage groups, 15 cM gap size, and the maximum likelihood objective function.

The locations of the markers in the physical (lentil v1.2 genome assembly) and genetic maps were compared and graphically represented using custom R scripts [60]. A circular comparative visualization of both maps was obtained using the R/Shiny application shinyCircos [61].

The interactive environment R/qtl [62] was used for mapping quantitative trait loci (QTL) onto the genetic linkage map by making use of Haley-Knott regression [63]. Genome-wide LOD significance thresholds were determined by a permutation test with 1,000 iterations at a significance level of 0.05. Confidence intervals were calculated using a 1.8-LOD drop method [64].

## Results

### Reference transcriptome, variant discovery, and segregation distortion

Sequencing of the 12 cDNA libraries from *L*. *culinaris* cv. Alpo yielded a total of 242,680,763 100-bp paired-end reads after the quality filtering. The normalization step retained 16,452,752 reads with an average input length of 200 bp, which were mapped onto the *L*. *culinaris* cv. CDC Redberry v1.2 genome assembly. The percentages of uniquely mapped, multimapped, and unmapped reads were 87.1%, 3.3%, and 9.6%, respectively. The normalized reads were *de novo* assembled using such mapping information, following the genome-guided method of the Trinity package, generating 61,194 ‘genes’ (Trinity’s definition) and 120,879 transcripts with an N50 of 2,521 bp. Duplicate and near identical contigs were removed in order to obtain a transcriptome from cv. ALPO that included 49,453 Trinity ‘genes’ and 102,892 transcripts with an N50 of 2,501 bp, an average length of 1,748.4 bp and a total of 179,931,148 assembled bases. When using only the longest isoform per gene the statistics amounted to an N50 of 2,219 bp, an average length of 1,308.2 bp and a total of 64,693,989 assembled bases. A total of 96.2% of the initially filtered reads aligned as proper pairs into the ALPO transcriptome. Transcript abundance was quantified and the single most highly expressed isoform for each gene was retained in order to obtain the reference transcriptome, containing 49,453 assembled sequences (S1 File) that were used later for the variant calling procedure.

**Fig 1:**
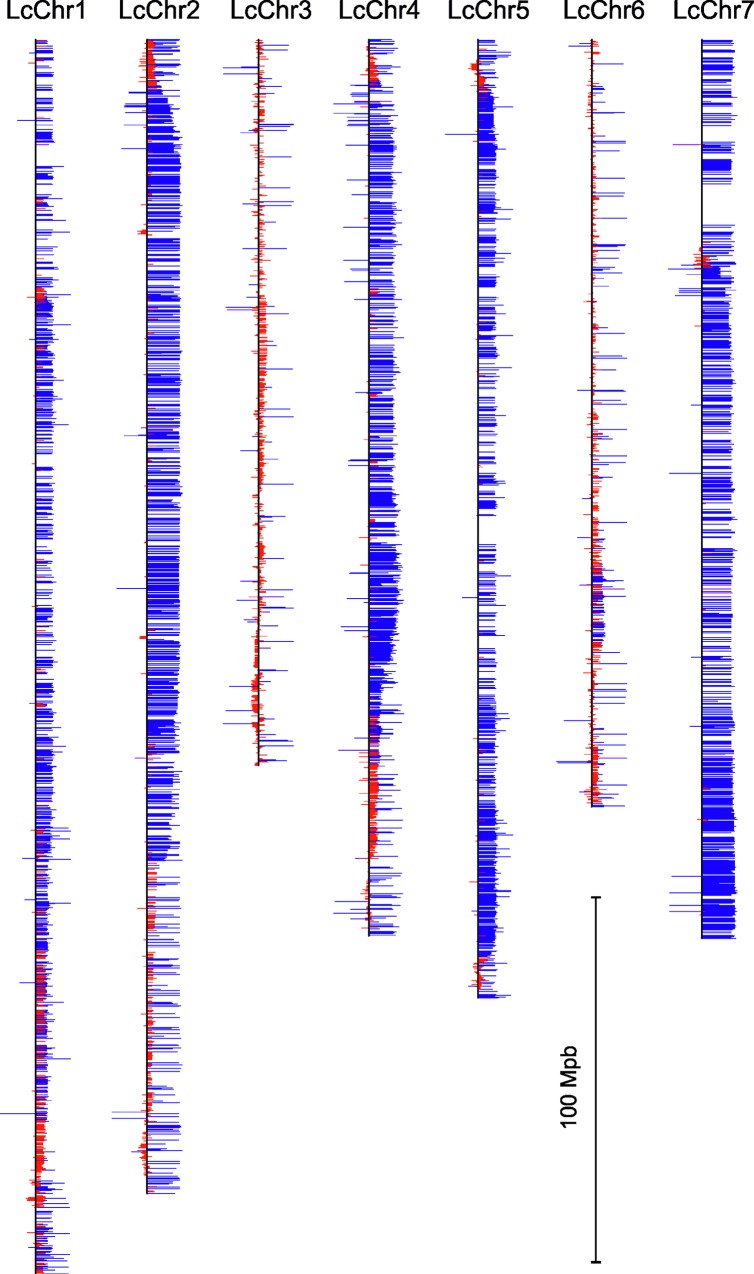
Location of the 13,128 polymorphic markers onto the seven pseudomolecules (LcChr#) of the *L*. *culinaris* cv. CDC Redberry v1.2 genome assembly. Size of horizontal lines represents the increased frequency deviation from the expected value of 0.5 (at vertical line) of the parental alleles derived from cv. ALPO (to the right) or *L*. *odemensis* (to the left). Blue horizontal lines denote distorted markers at p < 0.01 (chi-square test) and red horizontal lines denote markers used for the linkage analysis.

A total number of 1,139,915 variants (SNPs and short indels) were initially identified, and 120,483 were retained as quality polymorphic markers in the mapping population after undertaking the filtration applying the parameters described in the materials and methods section. These markers mapped onto 15,506 expressed genes. A single variant was chosen for each of them, applying the criteria of best adjustment to the expected 1:1 segregation considering the RIL genotypes. Most of these expressed sequences (13,128) were evenly distributed into the seven pseudomolecules (named as LcChr#) of the lentil v1.2 genome assembly, which correspond to the seven lentil chromosomes (Table 1; Fig 1). Another 1,996 sequences mapped onto minor contigs not yet assembled to the pseudomolecules and 382 sequences were not located in the lentil v1.2 genome assembly. Large regions were detected in LcChr2, LcChr4, and LcChr7 in which most of the marker frequencies scored near to the maximum for the allele derived from cv. ALPO (Fig 1). A higher than expected level of residual heterozygosity (average of 5.37%) was detected for the F_7_ interspecific RIL population (1.56%).

**Table 1:** Chromosomal distribution of the functional markers identified.

Chromosome^1^	Number of markers	Number of non-distorted markers^2^
LcChr1	2,137	688 (32.2)
LcChr2	2,227	797 (35.8)
LcChr3	1,619	1,462 (90.3)
LcChr4	2,014	736 (36.5)
LcChr5	2,089	470 (22.5)
LcChr6	1,284	961 (74.8)
LcChr7	1,758	131 (7.5)
LcContigs	1,996	924 (46.3)
Non-mapped	382	137 (35.8)
Total	15,506	6,306 (40.7)

^1^ Lentil genome assembly v1.2

^2^ Chi-square test, p < 0.01; percentages in parentheses

The chi-square test revealed that 5,676 markers did not deviate significantly (p > 0.05) from the expected Mendelian 1:1 ratio regarding parental allele contribution. The number of markers selected to construct the linkage map was increased up to 6,306 when the distorted makers that did not deviate significantly at p > 0.01 were included. These 6,306 markers were located on all of the seven chromosomes (pseudomolecules) of the lentil v1.2 genome assembly yet were unevenly distributed between and within them (Table 1). High densities of non-distorted markers were found all along of the LcChr3 and LcChr6, while they were concentrated at several regions of LcChr1, LcChr2, and LcChr4 or only at very specific regions of LcChr5 and LcChr7 (Fig 1).

### High-density linkage map and comparative analysis

**Fig 2:**
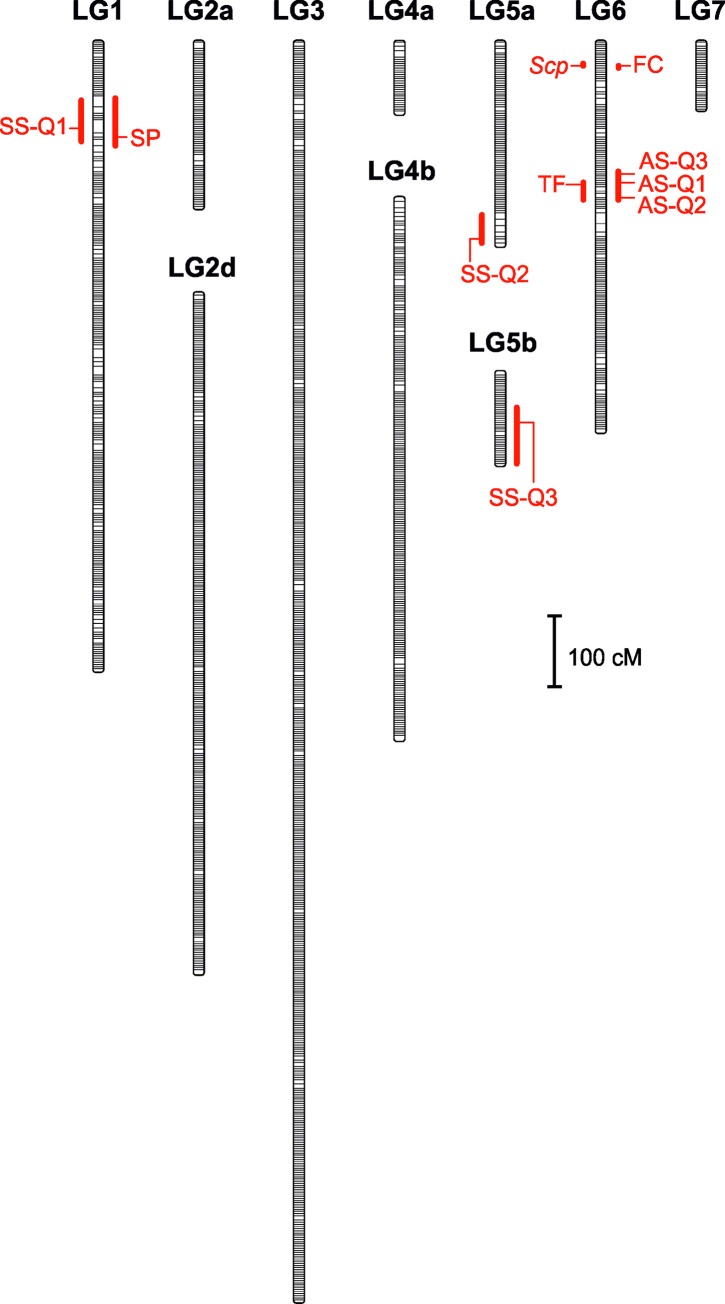
Linkage map of the RIL population derived from *L*. *culinaris* cultivar ALPO x *L*. *odemensis* accession ILWL235. Identified QTLs are shown as red horizontal lines (LOD peak) with solid vertical bars (with a 1.8-LOD drop confidence interval).

**Fig 3:**
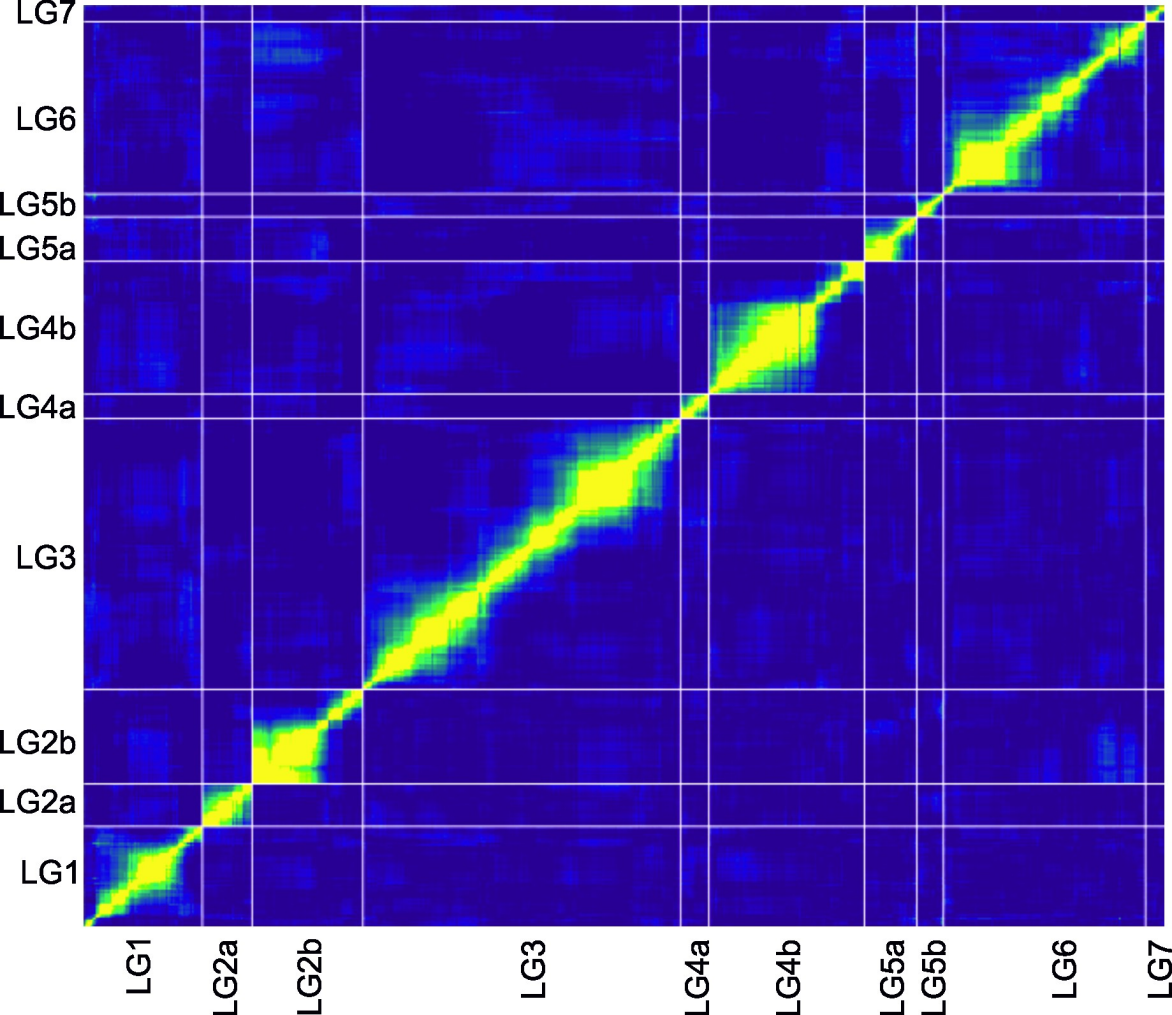
Heat plot for all of the linkage groups showing the pairwise comparison of recombination fractions (above of the diagonal) and the LOD score (below of the diagonal) for all of the markers uncovered. High recombination fraction estimates and low LOD scores are shaded in blue, while the warm colors represent the converse.

Data from the 6,306 SNP and short indels markers belonging to expressed sequences across of the 78 RILs were used to construct a high density linkage map (Fig 2). A total of 6,153 markers were mapped onto 10 linkage groups (LGs) while 153 markers remained unlinked to other markers (Table 2). These 6,153 markers grouped into 4,682 unique bins. The linkage groups were named using numbers and consecutive lowercase letters to match the respective lentil chromosomes of the lentil v1.2 assembly as best as possible. Marker name, linkage group and position (cM), reference transcript, and genome chromosome/contig location are provided in the S2 File. Mapping quality was checked using a heat map plotted for all the linkage groups wherein all the 6,153 markers were lined up against each other in order to evaluate the assignment to the linkage groups and the marker order (Fig 3).

**Table 2:** Marker distributions and genetic lengths of the linkage groups.

Linkage group	Number of markers	Number of unique bins	Length (cM)
LG1	678	597	889.49
LG2a; LG2b	283; 630	253; 623	232.78; 959.97
LG3	1,805	1,522	1,785.91
LG4a; LG4b	162; 884	79; 575	97.82; 760.53
LG5a; LG5b	297; 152	254; 122	284.76; 125.59
LG6	1,148	557	549.74
LG7	114	100	95.60
Unlinked	153		
Total	6,306	4,682	5,782.19

**Fig 4:**
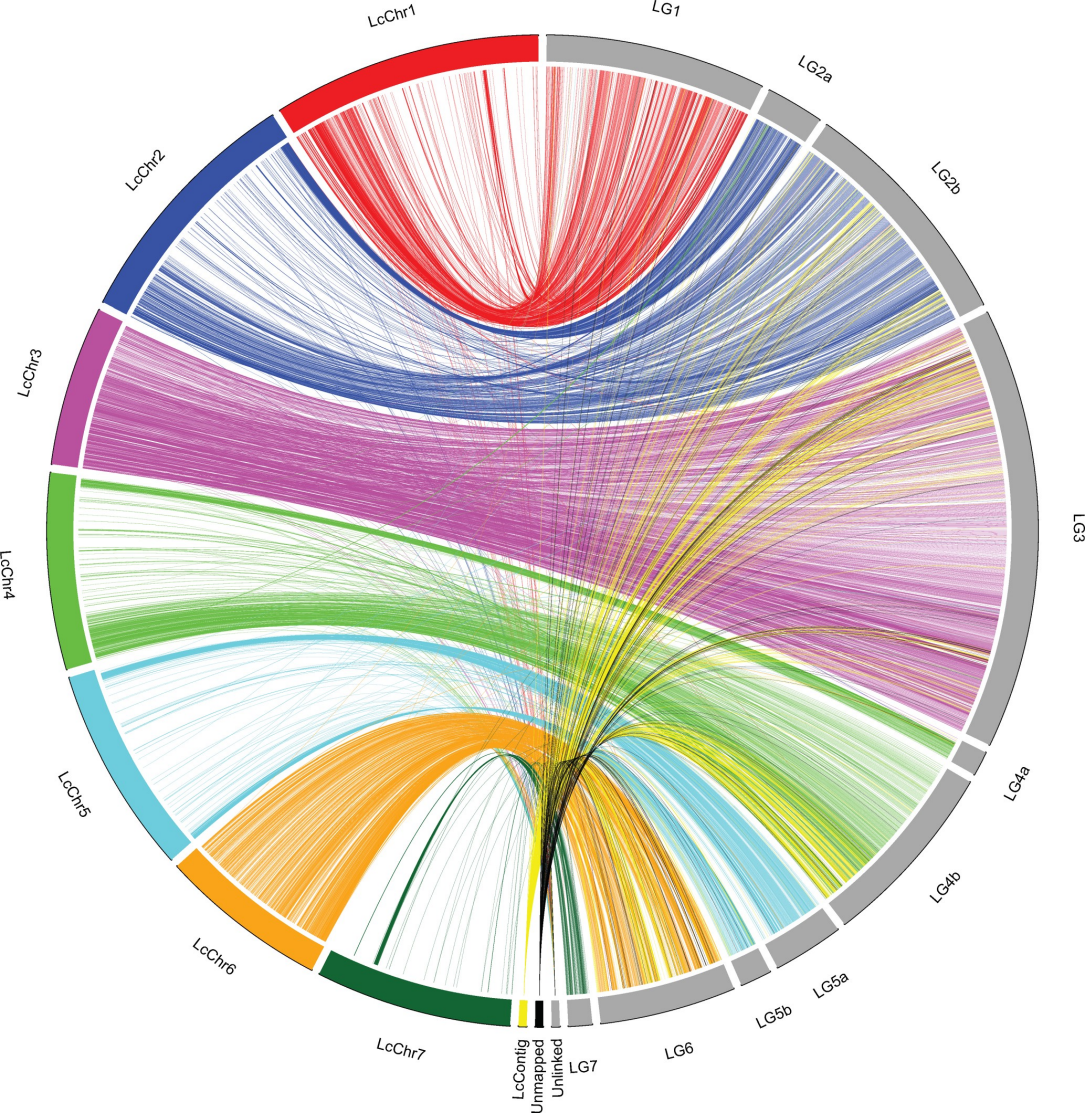
Circle comparative visualization of the markers locations in the lentil v1.2 genome and in the linkage groups of the RIL population genetic map.

**Fig 5:**
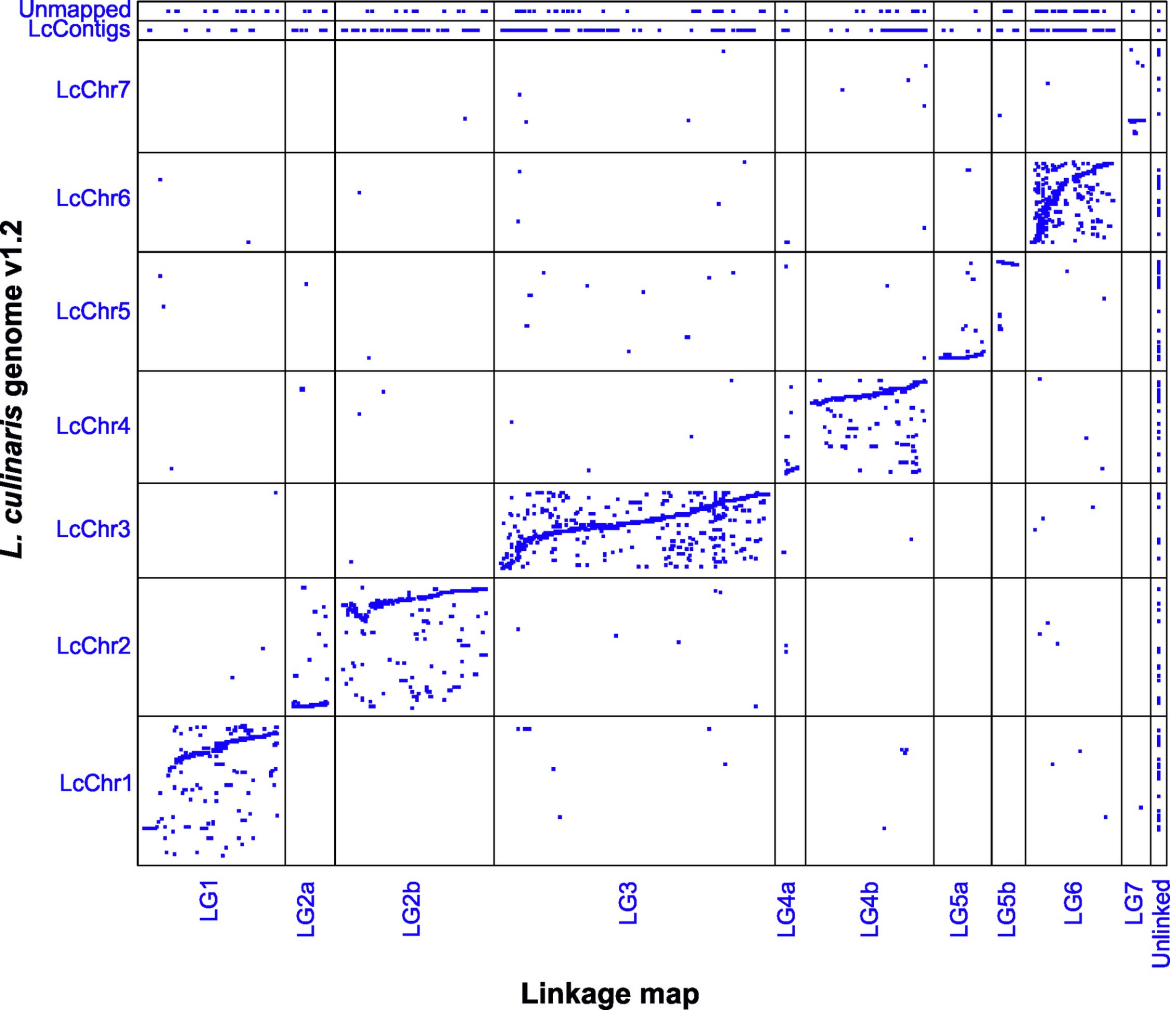
Dot plot representing marker collinearity between the lentil v1.2 genome chromosomes and the linkage groups (LGs) obtained from the genetic map constructed with the interspecific RIL population derived from *L*. *culinaris* cultivar ALPO x *L*. *odemensis* accession ILWL235.

The comparison of the high-density genetic map of the RIL population obtained from the interspecific cross *L*. *culinaris* x *L*. *odemensis* against the *L*. *culinaris* v1.2 genome assembly revealed a high level of collinearity for the location of most of the analyzed functional markers (Fig 4). Unlinked markers were distributed along all of the seven chromosomes and markers from genes that were not located onto the lentil v1.2 genome were present in different LGs that were assigned to all of the seven chromosomes according to the collinearity shown (Fig 5).

### Mapping morphological and agronomical traits

Two approaches were used to map three qualitative morphological characters: presence of seed coat spotting, flower color, and stem pigmentation. Firstly, by mapping the presence of related QTLs in the above linkage map (Fig 2) using the ‘scanone’ function of the R/qtl software with parameter model =“binary” to locate the binary characters as QTLs, in order to obtain a confidence region where the candidate genes could be located. Secondly, by constructing a new linkage map using the MSTMap software tool considering 6,309 markers by adding the binary encoded phenotypic data of the RIL population for each of the three qualitative morphological markers.

The character presence of seed coat spotting (*Scp*) showed a significant QTL located on LG6 (Table 3), accounting for 85.07% of the observed variability, with a peak at marker S044140 which was located in the gene Lc25388. This gene encodes a MYB transcription factor MIXTA-like protein which is annotated on LcChr6 at position 11,352,212. The markers included in the 1.8-LOD support interval (S2 File) belong to genes annotated in the genome between positions 11,352,212 and 14,634,141 of LcChr6, with the exception of gene Lc26377 (see below, as peak marker for flower color). This region of LcChr6 expands 3.3 Mb and currently includes 46 annotated genes. Character *Scp* was flanked by the QTL peak marker S044140 (gene Lc25388) and the S061401 marker (above-mentioned as gene Lc26377), when the obtained linkage map included data of the morphological markers.

**Table 3:** List of significant QTLs detected in the interspecific *Lens culinaris* cv. ALPO x *L*. *odemensis* ILWL235 RIL population.

Trait	QTL	LG^1^	Peak^2^	LOD	1.8-LOD^3^	Var.^4^	Add.^5^
Seed coat spotting (*Spc*; binary)	*Scp*	LG6	S044140	17.8	30.0–33.7	85.07	0.46
Flower color (FC; binary)	FC	LG6	S061401	16.5	31.3–34.6	84.20	0.46
Stem pigmentation (*Gs*; binary)	SP	LG1	S006811	6.0	79.0–145.7	33.96	0.32
Time to flowering (TF; range = 1–3)	TF	LG6	loc201	12.2	199.0–222.0	55.73	0.69
Seed size (SS; mm^2^)	SS-Q1	LG1	loc120	7.0	81.0–141.0	28.26	-1.28
range = 7.381–18.8	SS-Q2	LG5a	loc279	3.6	244.0–284.8	18.63	-0.97
mean = 14.89	SS-Q3	LG5b	S078816	3.2	47.0–125.6	23.13	-0.73
var = 4.02	Q1+Q2+Q3					35.48	
*Ascochyta* severity	AS-Q1	LG6	S046029 bin	5.4	180.0–222.0	27.14	-0.81
(AS; range = 1–7)	AS-Q2	LG6	loc219	5.0	180.0–222.0	25.53	-0.80
mean = 4.49	AS-Q3	LG6	S005268 bin	4.5	180.0–222.0	23.13	-0.73
var = 2.25	Q1+Q2+Q3					28.46	

^1^ Linkage group.

^2^ Marker or location (loc) in cM.

^3^ 1.8-LOD support interval in cM.

^4^ Phenotypic variance explained in percentage.

^5^ Additive genetic effect of the alleles derived from the parental accession *L*. *odemensis* ILWL235

The character flower color showed a significant QTL overlapping the one previously described for the *Scp* character on LG6 (Table 3) which accounted for 84.20% of the observed variability. The support interval included 37 markers distributed in six bins (S2 File). The corresponding genes for 25 of these markers are annotated in a 7.7 Mb region of the genome in which 106 genes are located between positions 11,352,212 and 19,096,974 of LcChr6 (which includes the complete 3.3 Mb region of the *Scp* QTL), nonetheless the peak marker S061401 is annotated at position 157,341,084 of LcChr6. This marker is located in the Lc26377 gene encoding for a phosphate transporter PHO1-like protein. Other nine markers from bins included in the support interval belong to genes also annotated out of the above range at LcChr6, including an anthocyanin 5-aromatic acyltransferase (gene Lc27646) at position 105,439,424. The trait flower color was flanked by the same markers than *Scp* (see above) in the linkage map obtained which included the data of the morphological markers.

The stem pigmentation character showed a significant QTL located on LG1 (Table 3) which accounted for 33.96% of the observed variability, with a peak at marker S006811 located in the Lc01979 gene (a Sulfite exporter TauE/SafE family protein) part of LcChr1 at position 75,787,823. The support interval included nine markers distributed in nine bins but two markers are currently annotated on LcChr5, one is positioned next to the peak marker, while another is located on LcChr6 of the draft genome assembly. Five out of the six LcChr1 markers are annotated between positions 46,253,696 to 96,580,293. This region of LcChr1 expands 50.3 Mb and counts with a total of 844 currently annotated genes. The character stem pigmentation was not included in any linkage group of the genetic map obtained considering also the data of the morphological markers.

QTLs displaying a significant association with the quantitative agronomical characters time to flowering (TF) and seed size (SS) were identified in the RIL population (Table 3). A single QTL was uncovered for TF on LG6, accounting for 55.73% of the observed variability, comprising a peak between the bins mapped at positions 199.47 cM (two markers from LcChr6 genes annotated between 152,135,041 and 155,170,846) and 203.37 cM (six markers from LcChr6 genes, four of them annotated between 151,824,573 and 155,170,846). The support interval included another seven markers in five bins, with a total of 10 markers annotated in a region of LcChr6 that expands 8.8 Mb (151,824,573 to 160,668,788) in which 157 genes are currently annotated. The *L*. *odemensis* parental accession contributed positive alleles that increased the time to flowering (Table 3).

Three QTLs accounting globally for 35.48% of the observed variability in seed size were mapped onto LG1 (SS-Q1), LG5a (SS-Q2), and LG5b (SS-Q3) (Table 3). The SS-Q1 support interval fully overlaps with the QTL identified for stem pigmentation at LcChr1 (see above) and its peak was located between markers mapped at positions 104.68 cM and 110.61 cM. The corresponding genes, Lc03317 and Lc01979, are annotated flanking the LcChr1 region spanning from position 75,787,823 to 96,580,293 (20.8 Mb) in which a total of 366 genes are annotated. The SS-Q2 peak was located between the markers mapped to LG5a positions 277.53 cM and 284.76 cM, which correspond to the genes Lc21173 and Lc23619, respectively, flanking the LcChr5 region from position 18,739,851 to 22,169,473 (3.4 Mb) which counts with a total of 118 currently annotated genes. The support interval included another four LcChr5 markers that expand the region up to 31.2 Mb (positions 15,801,723 to 47,019,826) holding 726 currently annotated genes. The SS-Q3 peak marker S078816 was located in gene Lc23140, an EF hand protein with potential roles in Ca2+ signal-mediated processes including growth and development [65], annotated at position 254,820,976 of LcChr5. The SS-Q3 support region included a total of 55 markers annotated in the LcChr5 region between positions 251,623,917 and 257,098,141 (5.5 Mb) with 204 currently annotated genes. The *L*. *odemensis* parental accession contributed negative alleles to seed size (Table 3).

### Mapping QTLs affecting resistance to *Ascochyta*

Three significant QTLs (AS-Q1, AS-Q2, and AS-Q3) which accounted globally for 28.46% of the observed variability to *A*. *lentis* resistance in the RIL population, were mapped within a common 1.8-LOD support interval onto LG6 (Table 3). The bin which contained the AS-Q1 peak included two markers from LcChr6 genes that are annotated between 139,108,707 (gene Lc27513, ATP-dependent helicase BRM) and 147,095,064 (gene Lc25909, Plant/F1M20-13 protein). The AS-Q2 peak was located between the marker mapped to position 214.09 cM (gene Lc27329, annotated at Chr6: 153,669,223) and the bin at 219.21 cM, which includes another three LcChr6 genes (Lc25078 at 108,049,280; Lc26336 at 152,733,327; Lc27664 at 154,983,742). The AS-Q3 peak was located in a bin that includes three LcChr6 genes at 79,397,962 (Lc25349, homeobox-leucine zipper ROC6), 140,610,179 (Lc25206, Methyltransferase PMT16), and 141,818,776 (Lc26436, uncharacterized protein). The 1.8-LOD support region included 22 bins containing a total of 75 markers located in LcChr6 genes plus 14 markers located in contigs not yet assigned to a chromosome. Most of the LcChr6 markers (69) are located in a 28.6Mb region between positions 132,032,337 and 160,668,788 which holds a total of 581 currently annotated genes; the remaining six markers were located in genes annotated in the following positions outside of those limits: 48,803,021, 53,893,775, 53,971,066, 79,267,292, 79,397,962, and 203,277,345. The *L*.*odemensis* parental accession contributed alleles that increased the resistance to *Ascochyta* infection as the scoring method [50] assigned low values to resistance and high values to susceptibility (Table 3).

## Discussion

High-throughput sequencing approaches, such as the RNAseq used in the present work, allow to generate and to characterize reference transcriptome datasets that support gene-based marker discovery useful and robust to undertake linkage mapping, trait dissection, or genetic diversity studies. Other transcriptome assemblies have previously been obtained with respect to other lentil cultivars using 454 Roche pyrosequencing [66,38], more recently also using the Illumina technology [67–69] and the Ion Proton system [70]. The assembly obtained in this work for cv. Alpo has displayed higherN50 values (2,501 bp), together with average (1,748.4 bp) and total lengths (179,931,148 bases) than those previously reported in transcriptomes obtained using Illumina sequencing platform. The N50 value is a metric widely used to assess the contiguity and the quality of an assembly [71]. Taking into account only the longest isoform of each gene, the total assembled bases amounted to 64,693,989 bp, corresponding to nearly half of the 130 Mbp of the whole lentil genome (4,063 Mbp) that has been identified as genic sequences [72]. The number of total assembled transcripts was also higher in the cv. Alpo transcriptome (102,879) than has been reported in previous publications (up to 84,069), while the number of different protein genes identified was lower (49,453) than the values reported by Singh et al. [68] which ranged from 52,565 to 55,667. Differences are a direct consequence of the distinct approaches that have been followed in the sequencing and read assembly processes, as well as a result of the different genotypes, plant tissues and development states of the materials studied.

Several genetic linkage maps of lentil (summarized recently by Ates et al. [36]) have been constructed using mapping populations mostly obtained by crossing cultivated lentil varieties or accessions. Intersubspecific maps developed from crosses using *L*. *culinaris* and the wild ancestor *L*. *culinaris* subsp. *orientalis* have likewise been published [73–75] while interspecific linkage maps have been limited to the case of *L*. *ervoides* [24]. The map presented here (*Lc*x*Lo*-map) is the first for an interspecific cross between the primary and the secondary lentil gene pools [33] based on *L*. *culinaris* and *L*. *odemensis*. The *Lc*x*Lo*-map is composed of 6,306 SNPs and short indels spanning a total of 5,782.2 cM with an average distance of 1.23 cM between adjacent marker bins. To date, this is the *Lens* sp. map that incorporates the largest number of markers, excluding a consensus map for *L*. *culinaris* that contains 9,793 markers obtained by integration of three component maps with 5,385, 4,177, and 2,439 markers [36]. It is worth mentioning that all the markers included in the *Lc*x*Lo*-map are located in different functional regions that are transcribed, instead of being distributed along the whole genome. Therefore, a higher average distance value between markers is expected compared to the low value (0.1 cM) reported in the case of the consensus map for *L*. *culinaris* based on DArT markers [36], because only 3,2% of the lentil genome is considered to hold functional genic sequences and gene density is not evenly distributed along the lentil genome [72].

The *culinaris x odemensis* map has been generated from a relative low number of available RILs and this fact could compromise the accuracy of the map, although a high collinearity level with respect to the location of the analyzed functional markers was found upon comparison against the lentil draft genome assembly v1.2.

The *Lc*x*Lo*-map has been used to find the genomic regions containing the candidate genes for three morphological characters. The seed coat spotting pattern (*Scp*) is an important characteristic that determines the market class and the end uses of the crop. A single gene controlling this trait has been proposed [41] and five alleles of the *Scp* gene have been reported, being the recessive homozygous genotype *scp scp* the non-spotted individuals [76]. The *Scp* locus was mapped onto LG6 by Fedoruk et al. [37] using an intraspecific linkage map with the same number of linkage groups as the seven lentil chromosomes, taken as the basis for the chromosome numbering of the lentil genome assembly [38]. The *Scp* locus was mapped by Eujayl et al. [77] to what they defined as LG3, with close linkage to the flower color locus. Kumar et al. [78] confirmed a monogenic complete dominance of purple flower color over white, fact previously proposed by Lal and Srivastava [79] who reported linkage with the mottled seed pattern.

We have found both traits, *Scp* and flower color (*FC*), in close linkage positioned in LG6 with overlapping support intervals for genomic location in the *Lc*x*Lo*-map. The *Scp* locus was mapped to a 3.3 Mb region of LcChr6 comprising 46 annotated genes including a candidate gene (Lc25388) encoding an MYB transcription factor MIXTA-like related with anthocyanin synthesis regulation and cell fate [80]. Most of the genes included in the *FC* support interval (25 out of 37) were annotated in a 7.7 Mb region that includes the whole 3.3 Mb region for *Scp*; nevertheless, another 10 genes which were included in the support interval are annotated outside of the 7.7 Mb region of the draft lentil genome, including gene Lc26377 corresponding to the flower color QTL peak marker. The Lc26377 gene encodes a phosphate transporter Pho-like that may have a pigmentation role as it has been reported that phosphate deficiency is related to anthocyanin accumulation [81,82].

Single gene inheritance of stem pigmentation in lentil has been reported with the purple color being controlled by a dominant allele [41,83–85]. Anthocyanin pigmentation of plantlet stems was present in the *L*. *odemensis* parental accession of the RIL population analyzed. This locus was mapped to LG1 using the QTL approach. However, the support interval included two genes currently annotated in LcChr5 and another gene annotated in LcChr6 of the draft genome assembly, all of them linked to five genes annotated in LcChr1 comprising a large 50.3 Mb region. The stem pigmentation locus *Gs* was mapped by Duran et al. [74] to their denominated LGIV, however an improved version of this map that our research group has obtained and which includes common markers with the map of Sharpe et al. [38], identified the named LGIV as part of the LG5/LcChr5 (unpublished data). The trait did not map to any linkage group when the map was obtained comprising the morphological markers. Therefore, it has not been possible to determine its precise chromosomal location using the *Lc*x*Lo*-map data.

A single QTL was identified for the character flowering time located on LG6. All the genes included in the support interval are located at the LcChr6 of the lentil draft genome v1.2, in an 8.8 Mb region encompassing a total number of 157 annotated genes. Using an intersubspecific cross of *L*. *culinaris* ssp. *culinaris* and *L*.*c*. ssp. *orientalis*, Fratini et al. [22] described a QTL for flowering time close to the *Scp* locus in a linkage group named LGI. As the *Scp* locus was mapped onto LG6/LcChr6 of the *Lc*x*Lo*-map (see above) near to the QTL position, these two QTLs found respectively in intersubspecific and interspecific mapping populations may be related. Recently and through marker-trait association analysis, Kumar et al. [86] identified two SSR marker loci associated with flowering time, each one explaining 15% of total phenotypic variation observed for this trait in a panel of 96 genotypes of cultivated lentil. We tried to locate these two markers (ALD 16 and ALD P5) in the lentil genome v1.2 using their available PCR-primer sequences [25]. The ALD P5 primers were located in the LcChr3 at a distance of 256 bp (from 83,245,520 to 83,245,775) while it was not possible to find the chromosomal position of the ALD 16 locus.

A total of three QTLs were detected for the seed size trait in LG1, LG5a, and LG5b, corresponding to one genomic location in LcChr1 and two in LcChr5. QTLs for seed size in lentil have been previously mapped by Fratini et al. [22], Fedoruk et al. [37], and Verma et al. [87]. The phenotypic variances of 37% and 60% reported by Fratini et al. [22] and Fedoruk et al. [37] for seed size QTLs are higher than that reported in our study (35.5%) and also to the 27.5% reported by Verma et al. [87]. The comparison of the QTLs identified by Fratini et al. [22] and Verma et al. [87] is difficult with respect to their map positions in the *Lc*x*Lo*-map because no common markers or linkage groups can be resorted to. Fedoruk et al. [37] identified three QTLs for seed diameter, the most important was located in LG1/LcChr1 around the cotyledon color locus (*Yc*). One of the *Yc* flanking markers was LcC17238p3606 that is located in position 202,217,501 of LcChr1. Using association mapping analysis with SNP makers of 138 cultivated lentil accessions, Khazaei et al. [88] validated two SNPs closely associated to the seed diameter trait, located in LcChr7 and LcChr1 which respectively explained 11% and 14% of the observed variation. The location of the SNP in LcChr1 (212,502,660) is relatively close to the *Yc* linked marker reported by Fedoruk et al. [37] and to one of the markers included in the support interval of the QTL detected in LG1 (163,405,141) of the *Lc*x*Lo*-map, with 242 genes annotated between them.

Several genetic linkage maps have previously been used to identify QTLs for *Ascochyta* blight resistance in lentil (reviewed by Rodda et al. [89]), with magnitudes varying from 3% to 89% of the phenotypic variance evidenced. Common QTL locations between different studies are difficult to establish because of the differing nomenclature for linkage groups and the lack of common marker loci between the genetic maps. In spite of that, the QTL named AB_NF1 on LG6 in the study of Sudheesh et al. [67] is comparable in position to QTL5 on LG1 of Rubeena et al. [85] and QTL1 on LG1 of Gupta et al. [49], based on a common SSR locus location [89]. The AB_NF1 QTL accounted for 7% of the phenotypic variation in an F_6_ RIL population from a cross of Indianhead x Northfield, being Northfield (ILL5588) a common parent in the majority of the studies published. We have located the SNP flanking markers of AB_NF1 in the genes Lc28181 and Lc25002 located in the LcChr6 positions 197,394,513 and 198, 226,847, respectively. Most of the markers included in the common support interval of the QTLs detected in LG6 of the *Lc*x*Lo*-map are annotated in the region extending from 132,032,337 to 160,668,788, however one of them is located in position 203,277,345. The genomic regions of AB_NF1 and the QTLs detected in this work are close enough to suggest that common genes may be involved in the *Ascochyta* resistance responses. Sudheesh et al. [67] reported that AB_NF1 linked markers were found to be associated with a minor gene that appeared to confer a partial resistance to an Australian field isolate (FT14125) evidencing on lentil genotypes a different pathogenicity pattern.

As new genetic diversity sources, wild relatives of crops have been used during many decades in plant breeding programs. Their utilization is expected to increase as a result of the ongoing improvements concerning species and diversity information and advances in breeding tools boosted by high throughput technologies. We have here reported the first genetic linkage map between *L*. *culinaris* and *L*. *odemensis* based on thousands of functional markers. This map has allowed to locate the genomic regions where the genes controlling different morphological and agronomical traits of lentil are present. Further studies in progress will allow for the precise identification of some of these genes.

## Supporting information

S1 File Reference transcriptome of cultivar ALPO. (TXT)

S2 File List of 6,306 markers used to construct the genetic map, ordered according to their respective linkage groups. (XLSX)
